# Language and Learner Specific Influences on the Emergence of Consonantal Place and Manner Features

**DOI:** 10.3389/fpsyg.2021.646713

**Published:** 2021-09-17

**Authors:** Yvan Rose, Natalie Penney

**Affiliations:** ^1^Department of Linguistics, Memorial University of Newfoundland, St. John’s, NL, Canada; ^2^Department of Communication Sciences and Disorders, University of Maine, Orono, ME, United States

**Keywords:** phonology, emergence, phonetics, phonological features, lexicon

## Abstract

This article focuses on the emergence of consonantal place and manner feature categories in the speech of first language learners. Starting with an overview of current representational approaches to phonology, we take the position that only models that allow for the emergence of phonological categories at all levels of phonological representation (from sub-segmental properties of speech sounds all the way to word forms represented within the child’s lexicon) can account for the data. We begin with a cross-linguistic survey of the acquisition of rhotic consonants. We show that the types of substitutions affecting different rhotics cross-linguistically can be predicted from two main observations: the phonetic characteristics of these rhotics and the larger system of categories displayed by each language. We then turn to a peculiar pattern of labial substitution for coronal continuants in the speech of a German learner. Building on previous literature on the topic, we attribute the emergence of this pattern to distributional properties of the child’s developing lexicon. Together, these observations suggest that our understanding of phonological emergence must involve a consideration of multiple, potentially interacting levels of phonetic and phonological representation.

## Introduction

The sound systems of human languages are usually described in terms of speech sounds (consonants, vowels) and their phonological features, for example the [oral]∼[nasal] contrast displayed pairs of sounds such as [b] and [m], which encode meaning differences between words such as *bat* ∼ *mat*. In the tradition of Jakobson (1941) and Trubetzkoy (1969), phonological features are considered the smallest, most atomic units of language. More controversial is the question as to where features come from. Nativist models of generative linguistics assume that linguistic primitives such as features are innately available to the learner (Chomsky, 1957; Chomsky and Halle, 1968; Smith, 1973; Hale and Reiss, 2003, 2008). However, this view has been challenged in recent years for its failure to predict that similar consonants and vowels, which can be described using identical sets of phonological features, may pattern phonologically in very different ways across languages. Another key observation is that morpho-phonological patterns do not always follow expectations based on properties of speech phonetics (Mielke, 2008, 2013; Cowper and Currie Hall, 2014; Dresher, 2014, 2018). For example, classes of sounds such as laterals and nasals may display drastically different behaviors across languages (e.g., laterals patterning as stops or as continuants; nasals patterning as voiceless or voiced consonant; Rice, 1993; Mielke, 2005a). Observations such as these strongly suggest that phonological feature specification must emerge on language-specific grounds, and that speech phonetics cannot be taken as the sole source of the patterns observed.

On the other side of the theoretical spectrum, these same observations have been taken as arguments toward (self-termed) “radical” views of phonology which, in the tradition of Waterson (1971), reject the hypothesis that phonological features even exist as psychologically real units of representation (Vihman and Croft, 2007; Ambridge, 2020). Within these models, phonological processing takes place over whole-word units memorized within the lexicon, and every explanation stems from functional mechanisms such as analogy, where factors such as auditory perceptibility, articulatory complexity and usage frequency also play a central role in shaping phonological behaviors (e.g., Bybee, 2001). These models are thus poorly equipped to capture the emergence of phonological patterns affecting particular sounds or classes of sounds. For example, stopping is a production pattern in child language which typically affects sound classes such as fricatives across different places of articulation (e.g., *fun* |ˈfʌn| → [ˈpʌn]; *sun* |ˈsʌn| → [ˈtʌn])^1^. This pattern can be captured by models that relate these sounds through the relevant features they share (here, a manner feature such as [continuant]), independent of specific places of articulation such as [labial] or [coronal]. In word-based models, such analyses are not possible, because phonological features are immaterial and, from a phonetic perspective, labial and coronal sounds involve their own phonetic cues, speech organs and related motor plans. These models also fail to capture the uniform application of patterns across different word forms; while the two words above could be related for their being CVC in shape with an initial fricative, this word-based analysis comes short of capturing similar patterning in words like *casino* |kəˈsino| produced as [kəˈtino] by the same learners. An outright rejection of phonological features is thus tantamount to throwing out the phonological baby with the theoretical bathwater, as it immediately limits our ability to capture and, ultimately, understand patterns of phonological development robustly attested within the literature (Rose, 2014, 2020).

In light of this, theories of phonology which build on segmental units (i.e., speech sounds and their phonological features), prosodic domains (e.g., syllables, metrical feet) and interactions between these different levels of representation are much better equipped to capture phonological patterning in a cohesive fashion (Selkirk, 1980a, b; McCarthy and Prince, 1986). However, these representational theories of phonology tend to focus more on the units and domains needed to explain phonological behaviors than on the origins of these units.

This is where emergentist models of phonology and phonological development become centrally relevant. According to these models, abstract categories are real units of representation but are not innate. In a nutshell, emergentist models which do embrace abstract categories such as phonological features share the hypothesis that language learners identify units of speech present in the ambient language and make generalizations about the distributions of these units within and across different prosodic and/or lexical domains. It is these generalizations that form the basis for the emergence of abstract segmental and prosodic categories in the learner’s mental representations of these words (e.g., Pierrehumbert, 2003; Goad and Rose, 2004; Lin and Mielke, 2008; Menn et al., 2009, 2013; Munson et al., 2011; McAllister Byun et al., 2016; Rose et al., 2021):

On this understanding, the system of phonological categories includes not only segments, but also other types of discrete entities in the phonological grammar, such as tones, syllables, and metrical feet. Each of these [categories] has phonetic correlates in its own right (Pierrehumbert, 2003, p. 119).

**FIGURE 1:**
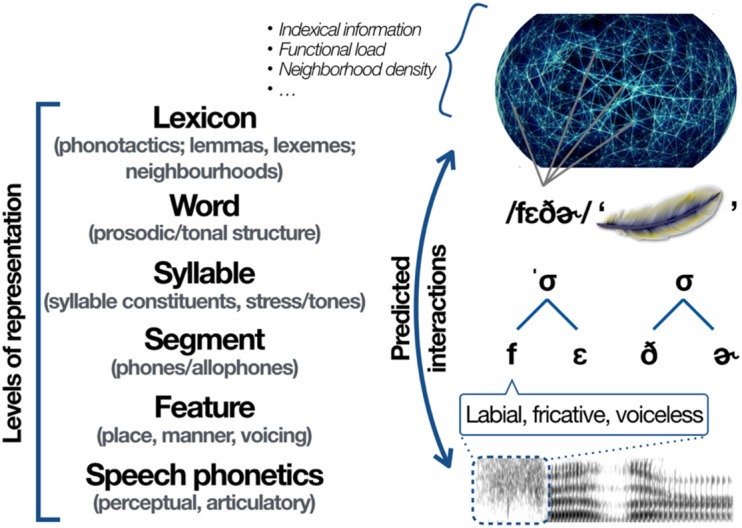
General model of phonology, from speech phonetics to the lexicon.

Building on this general hypothesis, we assume, in the discussions that follow, the general model in Figure 1. We claim that both the emergence of the categories represented at each level of this model as well as their presence in the learner’s system after they have emerged have the potential to influence aspects of phonological development (see also Menn et al., 2021).

We support our argument through the study of phonological patterns that make reference to three main levels of representation, specifically the sub-segmental (phonological features), segmental (speech sounds) and lexical (word-size units) levels. We draw on systematic observations extracted from cross-linguistic data available through the PhonBank database (^2^Rose and MacWhinney, 2014). We first study the acquisition of rhotic consonants (“r” sounds) across languages, and show that not only the phonetics of these rhotics must be considered to understand the patterns observed, but also the larger system of phonological contrasts and related phonetic properties displayed by each language. We then engage with a second study, this time focusing on early word productions by a single learner of German. This child uses the labial place of articulation in his early attempts at coronal consonants which involve continuancy, however, only in word onsets; these coronals do not undergo labial substitution in non-initial positions. In order to account for this pattern, we build on influential work by Fikkert and Levelt (2008) concerning how child phonological patterns might originate from pressures coming from the phonological content of the learner’s own lexicon.

As noted by one reviewer, the relation between the two studies detailed below may not seem obvious at first, given that the first study consists of a cross-linguistic survey of segmental development, while the second focuses on an individual learner’s acquisition of a particular class of sounds. However, it is through combining these two studies within a single discussion that we can highlight predictions made by encompassing models of phonological emergence such as that in Figure 1 concerning emergence within different levels of representation as well as potential interactions across these levels.

## Data and Methodology

For our first study, we considered longitudinal data collected in naturalistic settings from 30 children documented across four different languages (Dutch, French, German, and Portuguese). Our main inclusion criterion was that the children had not already acquired the uvular rhotic of their language at the beginning of the observation period documenting the development of their speech productive abilities. Age differences between participants at the onset of meaningful speech or at the time when they began to produce uvular rhotics accurately are thus largely irrelevant to the data descriptions and comparisons below. The Dutch data include 9 children from the CLPF corpus (Fikkert, 1994; Levelt, 1994), recorded between the ages of 1;0 and 2;11. The French data were collected from four different corpora documenting 9 monolingual learners between the ages 0;11 and 6;11: Goad and Rose (Rose, 2000, 2003), Lyon (dos Santos, 2007; Demuth and Tremblay, 2008), Paris (Morgenstern and Parisse, 2007; Leroy-Collombel et al., 2009), and Yamaguchi (Yamaguchi, 2012, 2015). The Portuguese data are from 8 learners documented within the CCF and Freitas corpora^3^, recorded between the ages 0;7 and 4;10 (Freitas, 1997; Correia, 2009; Correia et al., 2010; da Costa, 2010). Finally, the German data are from the four learners of the Grimm corpus, who were documented between the ages of 1;0 and 2;1 (Grimm, 2006, 2007).

To analyze these data, we employed the query and analysis functions built into the Phon software program (^4^Rose et al., 2006; Rose and MacWhinney, 2014), which provides useful methods to capture segmental behaviors across phonologically determined positions. We focused primarily on word-initial, singleton onset consonants, in order to control for distributional differences between languages (e.g., Portuguese does not allow for |ˌ| in syllable codas) and issues related to the development of consonant clusters. When relevant, we included observations from non-initial onsets for comparison purposes. Toward the analysis of word-initial consonants, we ignored segmental deletions resulting from full syllable truncation, such that words like <*gi*>*raffe* “giraffe” and <*ge*>*macht* “made” were treated as *r*- and *m*-initial, respectively. The truncation in <*gi*>*raffe* can be attributed to the fact that the initial syllable in this word is unstressed and, as such, arguably missing from the child’s early phonological representation for this word (e.g., Demuth, 1995; Gerken, 1996; Jusczyk et al., 1999; Mattys et al., 1999; Grimm, 2007). In a similar way, truncation of the verbal prefix *ge* in <*ge*>*macht* can either be the result of it being unstressed, similar to the initial syllable of <*gi*>*raffe*, and/or arise from the fact that this morphological marker was arguably not yet acquired by the learner, as evidenced by the fact that Wiglaf systematically failed to produce this morpheme during the period relevant to the current study.

We generated developmental timelines for each child and made observations about the places and manners of articulation of the consonants they produced. For example, the German word *loch* |ˈlɔx| “hole” produced as [ˈvɔx] displays a coronal-to-labial place substitution. Such substitutions, in addition patterns of deletion and accurate production, are at the center of our descriptions in the ensuing sections.

## Cross-Linguistic Survey on the Development of Rhotic Consonants

We begin with our survey of the development of rhotic consonants across languages. As we will see, learners of different languages may take markedly different paths in their development of phonetically similar sounds. Before we engage with these data, we summarize, in the next section, information about the phonetics and phonology of rhotic consonants across languages.

### Typological Observation and Predictions

Most of the world’s languages display rhotics as part of their consonantal inventories (Maddieson, 1984, p. 73). Rhotics also share several commonalities across languages, for example their widespread distribution as part of onset and coda clusters in languages which allow for such clusters^5^. This similarity in phonological distribution is remarkable given the rather extreme range of phonetic variants in which rhotics express themselves across languages. For example, Dutch (van de Velde and van Hout, 2001; Scobbie and Sebregts, 2011), German (Wiese, 1996, 2003) and French (Ostiguy and Tousignant, 1993) all display uvular continuants which range phonetically from more or less devoiced fricatives to fully voiced trills [ʀ, ʁ, χ]. Each of these languages also display a wide range of non-uvular rhotics across their regional dialects, however, without significant consequences for the phonological patterning of these rhotics (Ladefoged and Maddieson, 1996, p. 215). For example, uvular rhotics display virtually the same distributional properties in syllable onsets as the apical flap or tap of languages such as Portuguese^6^ and Spanish or the retroflex approximant of English^7^. There is thus a relative disconnect between the highly variable phonetics of rhotics within and across languages and their generally stable phonological patterning across these same languages.

Models of segmental representation in the tradition of Jakobson (1941) and Trubetzkoy (1969), which build on cross-linguistic typological evidence, uniformly capture this disconnect between the phonetics of rhotics and their phonological patterning through abstract (phonological), as opposed to concrete (phonetic) features. The obvious start under this view is the observation that the different languages have different set of phonemes, whereby neither French nor Portuguese displays |h| in their inventories, in contrast to Dutch and German. However, as we discuss below, this observation alone falls short of explaining the source of the segmental knowledge acquired by the child learners which yielded the different behaviors observed across languages.

Models that assume innate categories (e.g., Hale and Reiss, 2003, 2008) must explain both the selection of given phonetic substitutes as well as the fact that the same substitutes appear to never be available, for phones that are essentially the same, for learners of other languages. However, because these models generally abstract away from issues in speech phonetics, they are not very well equipped to predict different patterns of substitution for different types of rhotics, or whether similar consonants should display similar developmental patterns across learners of different languages. We indeed want a model which can predict developmental trajectories within individual languages, and also determine to what extent we can compare trajectories between phonologically similar but phonetically different segments. Beyond theoretical modeling, these questions also have clear clinical and educational implications, for example concerning the diagnosis and treatment of speech disorders, especially in the context of languages for which there are no established norms for speech sound acquisition (e.g., McLeod and Crowe, 2018).

By comparison, emergentist models have the potential to offer a more detailed developmental picture, as they must consider units of speech in light of both their phonological *and* phonetic properties. This is the essence of both the Linked-Attractor model (Menn et al., 2009, 2013) and the A-map model of phonological development (McAllister Byun et al., 2016), both of which explicitly relate auditory perception and articulatory production, both of which demonstrably vary on language-specific grounds (Pierrehumbert, 2003), to the emergence of segmental representations. We return to this discussion after we introduce the relevant evidence, in the next subsection.

### Rhotic Development Across Languages

Table 1 presents general trends in the acquisition of uvular rhotics (henceforth referred to as |ʀ|) across learners of Dutch, French, German, and Portuguese. Two inter-related observations emanate from these data. First, while noticeable percentages of [h] substitution for |ʀ| are recorded for Dutch and, in particular, German, only very marginal traces of this pattern are found in French and Portuguese. Second, these latter languages display noticeably more prominent patterns of |ʀ| deletion.

**TABLE 1:** General trends in the acquisition of |ʀ| in singleton onsets across four languages.

Language	# children	|ʀ| attempts	[h] substitution	%	(range)	|ʀ| deletion	%	(range)
**Dutch**	9	1,693	347	19.7%	(2.1 – 42.2%)	334	20.5%	(6.3 – 36%)
**German**	4	1,000	307	30.7%	(15.2 – 47.2%)	201	20.1%	(13.6 – 26.4%)
**French**	9	4,034	6	0.1%	(0 – 0.5%)	2,234	33.3%	(8.5 – 80.1%)
**Portuguese**	8	966	4	0.4%	(0 – 0.8%)	381	39.4%	(11.2 – 59.4%)

That |ʀ| deletion is attested during early stages across all four languages is expected, given widespread deletion patterns, observed among all child language learners, at the stages when they have not yet attained a motor plan to reproduce given sounds^8^. More important is our observation that French and Portuguese learners generally move from deleting |ʀ| to producing it in an adult-like fashion. In contrast to this, [h] substitution as an intermediate stage is well attested in the productions of both Dutch and German learners, even if it cannot be considered a necessary stage of development (4 of the 9 Dutch children transitioned more directly from deleting |ʀ| to producing it accurately)^9^.

**FIGURE 2:**
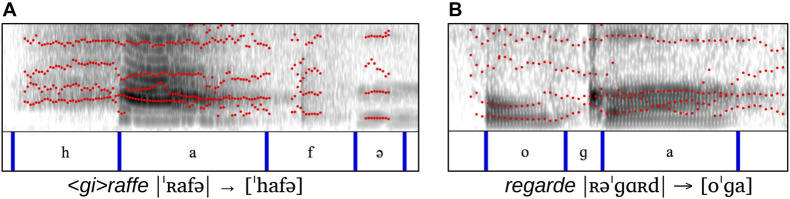
Illustrations of [h] substitution vs. |ʀ| deletion. **(A)** *<gi>raffe* |ˈʀafə| → [ˈhafə]. **(B)** *regarde* |ʀəˈɡɑʀd| → [oˈga].

In Figure 2, we provide representative spectrograms to illustrate [h] substitution and |ʀ| deletion. The example in Figure 2A comes from a production of <*gi*>*raffe* |ˈʀafə| “giraffe” by German-learning Wiglaf, who truncated the first (unstressed) syllable and substituted [h] for |ʀ|^10^. As we can see, [h] figures prominently, also with noticeable duration, in word-initial position, where it occupies the place of target |ʀ|.

This differs clearly from the form in Figure 2B, by French-learning Anaïs, whose production of the word *regarde* |ʀəɡɑʀd| “look (imp.)” undergoes initial |ʀ| deletion (in addition to word-final cluster deletion), with only background noise, as opposed to [h], preceding the initial vowel.

**FIGURE 3:**
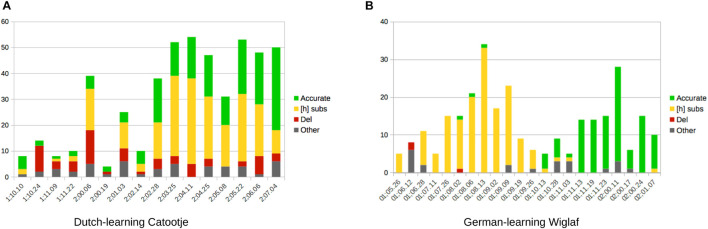
Variable vs. categorical behaviors in the emergence of |ʀ|. **(A)** Dutch-learning Catootje. **(B)** German-learning Wiglaf.

While the pattern of |ʀ| deletion clearly stands out of our survey of French and Portuguese, that of [h] substitution observed in Dutch and German is itself more variable. First, [h] substitution is not attested to the same extent in the productions of all of the children learning these latter two languages. Second, when this substitution occurs in noticeable amounts in the speech of individual learners, it can present either categorically or more variably. In the latter case, [h] substitution may alternate with |ʀ| deletion and/or production, at times over extended periods of development. Figure 3 illustrates this developmental difference. As we can see in Figure 3A, Catootje alternated between [h] substitution, |ʀ| deletion and |ʀ| production over a period of approximately 9 months. A further look at the data for this child also shows that the variation cannot be attributed to particular words or word forms.

In contrast to this, in Figure 3B, Wiglaf’s development of |ʀ| was much more rapid, and also characterized by a period where [h] substitution was the clearly dominant pattern, before the child mastered the production of |ʀ|. Further, the few transcripts which display noticeable exceptions to the leading patterns identified in the chart also reveal alternative productions which are extremely close to the leading pattern at each stage. For example, all but one of the “other” productions which occur early in the corpus (1;06.12 – 1;06.28) involve substitution by [ʔ], another laryngeal consonant, making this outcome very comparable to the leading pattern of [h] substitution observed during this period. Likewise, the substitutions observed during the 1-week period between 1;10.28 and 1;11.03 as well as sporadically in later sessions almost all involve substitutions to [x] and [ɣ], both of which are, from an articulatory standpoint, extremely close to the target rhotic, whose accurate production became the clearly dominant pattern during the subsequent 10-day period.

While studying cross-linguistic or individual variation for [h] substitution in more detail transcends the scope of this article, we take the different trends observed in our survey as predictable under emergentist approaches. The more categorical segmental behaviors point to representations fully phonologized by the learner, while the more variable ones, which tend to be more prominent during the very early stages of segmental emergence or, later, during transitions between stages, suggest representations not fully firmed up within the learner’s system. This can be due to misleading variation in the auditory signal, or the children’s imprecise mappings of the auditory categories into articulatory categories and related gestures needed for the reproduction of these units in speech.

Given the phonetics of |ʀ|, a uvular rhotic whose cues to place and manner of articulation are rather elusive, it is not surprising to see deletion as a noticeable pattern during early stages across all four languages. The consonant presents as a subtle constriction around the uvula, resulting in a trill, a fricative, part of which also depends on the degree of voicing, which also often varies between languages or language dialects (Ladefoged and Maddieson, 1996, p. 167). Until the learner attains even the most basic way to reproduce uvular rhotics, they must perform relatively complex analyses of the auditory signal for this consonant, also in the absence of obvious visual cues, given the location of the uvular place of articulation at the back of the oral cavity. In turn, the reproduction of these cues in speech involves the fine-tuning of controlled articulations such as the partial raising and backing of the tongue dorsum, subtle constrictions of the velopharyngeal area, combined with the particular aerodynamic control of the more or less phonated (voiced) airflow making its way through these constrictions (Ohala, 1983), the detail of which also depends on the precise realization of the uvular rhotic as a fricative, a trill, or anything in between (Ladefoged and Maddieson, 1996, p. 225).

Also key to our argument about emergence is the virtual absence of [h] substitution in French and Portuguese. Recall the general emergentist hypothesis that learners build their phonological representations in part from their analyses of the phonetic dimensions that define the ambient language. We suggest that it is the presence of the laryngeal fricative |h| in Dutch and German, and the absence of this consonant, and of the phonetic space it defines, in French and Portuguese, which sets the cross-linguistic difference highlighted in Table 1.

An reviewer offered a potential counterpoint to this second claim, namely that [h] substitution may not be possible in languages that do not display this or similar sounds (e.g., [ɦ, ʜ]) in their inventories. We agree with the broad strokes of this analysis. We, however, see it as limited in that it only offers a partial picture of the facts, for it lacks a mechanism to actually limit the learner’s exploration of potential substitutes for the sounds present in the ambient language. Indeed, analyses which do not address the origins of phonological categories are left with the double problem of explaining why patterns of substitution happen in some languages while they are virtually never attested in other languages. Further, this broad analysis would fail to account for more subtle effects seen in our data, especially between Dutch and German, which do point to a relative, rather than absolute, prediction about developmental patterning across languages. We can indeed relate the relatively lower percentage of [h] substitution as well as the higher rate of |ʀ| deletion in Dutch, in comparison to German, to the fact that in the German dialect of the children documented within the Grimm corpus, the voiced/voiceless contrast among plosive obstruents is best described as degrees aspiration, or positive voice onset time (Kleber, 2018, and references therein), while Dutch displays voicing contrasts more comparable to that of French or Portuguese, whereby voiceless stops are generally plain (unaspirated) and voiced stops display a degree of pre-voicing, or negative voice onset time (Lisker and Abramson, 1964; van Alphen and Smits, 2004). German thus displays more robust aspiration cues than that of Dutch, hence the more robust pattern of [h] substitution in the German data revealed by our survey. Finally, neither French nor Portuguese displays [h] in its inventory or aspiration cues in its expression of voicing contrasts. These languages thus lack the phonetic categories and, by extension, the phonological representations that could compel the learner toward laryngeal substitutes, making [h] substitution for |ʀ| unlikely in these languages.

### Interim Discussion

These observations have implications for both word-based and nativist views of phonology and phonological features. On the one hand, word-based approaches view phonological development as the child’s approximation of the phonetic properties of whole-word forms. If this were the case, then patterns of [h] substitutions could be expected for French and Portuguese as well, given the overall phonetic proximity between uvulars and laryngeals (also with a range of potential pharyngeals in between). From a strictly analytic perspective, even our descriptions above (as well as in the next section) are irrelevant to these approaches, given that segmental or subsegmental patterning can neither be predicted nor analyzed within frameworks that reject segments and features in the first place. On the other hand, as discussed already, nativist theories that rely on a universal set of features lack the level of phonetic specificity required to capture our observations above. This second point can also be reinforced if we consider patterns of rhotic development in additional languages. For example, in Portuguese and Spanish, substitutions for the apical tap |ɾ| and trill |r|, both of which are generally late-acquired, yield substitutions to [j] or [l] in a majority of reported cases where children produce continuant substitutes for these rhotics (Goldstein, 2007; da Costa, 2010). This is consistent with the general phonetic properties of these consonants (e.g., coronality, sonorant continuancy). Similarly, the rhotic approximant |ɹ| of English presents labialized [w] substitutions as the overwhelmingly predominant pattern (Smit, 1993), especially in pre-vocalic (onset) positions^11^. Given that |ɹ| involves dimensions within the auditory space characterized by a lowering of the third formant, itself enhanced by variable degrees of lip rounding (Stevens and Keyser, 2010; Ladefoged and Johnston, 2011), the auditory and articulatory overlaps between these two sounds make [w] a ready substitute for |ɹ| (see, also, Roberts, 2019, for a discussion of these issues based on an acoustic study of |ɹ| development).

We add to these observations the recent survey of the development of rhotic taps and trills across seven different languages (Bulgarian, Hungarian, Icelandic, Portuguese, Slovenian, Spanish and Swedish) by Bernhardt and Stemberger (2018). In line with our results above, this survey reveals cross-linguistic differences in the acquisition of phonologically similar rhotics, and many of these differences cannot be accounted for based on phonological features alone. As these scholars put it: “[w]e cannot rule out the possibility that the /r/ is articulated in subtly different ways in different languages and that those subtle differences lead to interactions with structural complexity” (Bernhardt and Stemberger, 2018, p. 568). We fully concur with this statement, which also calls for a re-examination of the cross-linguistic differences observed in this survey in light of both the language-specific phonetics of each rhotic and the overall phonetic and phonological properties of each of these languages (e.g., Ladefoged and Maddieson, 1996 for a starting point; a cross-language acoustic and/or articulatory study of rhotic productions would offer compelling new evidence).

On a related note, the literature on covert contrast suggests that at least a portion of substitutions such as those reported above may be misleading, given that adult transcribers often perceive two different phonetic outcomes produced by a child (e.g., “true [w]” and “labialized [ɹ]” both perceived as a single “[w]” category; e.g., Macken and Barton, 1980; Scobbie et al., 1996; Munson et al., 2010; Richtsmeier, 2010; see, also, Roberts, 2019, and Rose et al., to appear, for recent discussions). We concur that such effects may have affected some of the transcription data we used for this article. For example, as mentioned already, most of the alternate substitutions reported in Figure 3B for Wiglaf are phonetically close to the child’s leading pattern at the time they were recorded. It is thus possible that some of the child’s productions were straddling the transcribers’ perceptual boundaries between these closely similar phonetic alternatives. We leave this eventuality open for further research based on acoustic measurements of the relevant speech samples. In spite of these additional questions, our general argument about phonological emergentism holds fully, that predicting actual patterns of production for particular sounds must involve a consideration of both the system of contrasts and the phonetic expression of these contrasts in each relevant language.

In this context, Bernhardt et al. (2015), who compare the development of fricatives by English, German and Icelandic learners, observe that English and German children use affricate outputs more prominently than Icelandic children do. These scholars relate this observation to the absence of the phonological feature relevant to affrication in Icelandic, given that this language, as opposed to English and German, does not display affricates in its inventory. The emergentist approach we advocate for in this paper is very close to this in spirit, but also offers a mechanism to address the origin (or absence, in the case of Icelandic) of the relevant units of phonological representation: In the absence of affrication within the Icelandic auditory space, Icelandic learners have no reason to develop an articulatory mapping for affricates and, as such, are unlikely to make systematic use of these consonants as substitutes for other sounds in their speech productions.

Finally, it is important to stress that while, under the current view, phonetic factors play a prominent role in explaining patterns of segmental development, there are also clear limits on what can be explained through speech phonetics. Categorical behaviors influenced by units of different sizes indeed pervade the literature on child phonology, many of which, for example at the level of syllable and metrical structure, transcend predictions that can be achieved based on phonetic factors (e.g., Smith, 1973; Fikkert, 1994; Barlow, 1997; Freitas, 1997; Rose, 2000; Inkelas, 2003; Gnanadesikan, 2004; Goad and Rose, 2004; Goad, 2006; Rose and dos Santos, 2010). As mentioned above, we take both the emergence of segmental units and their later interactions within the learner’s system as sources of explanation for phonological development.

In the next section, we keep our focus on segmental substitutions, but discuss how these may also arise from other aspects of the learner’s developing system, in particular the phonological knowledge encoded at the level of the lexicon.

## Lexical Pressure on Phonological Development

We now turn to the productions of an individual learner of German, Wiglaf, from the Grimm corpus introduced in see section “Data and Methodology.’ Between the ages of 1;08.02 and 1;10.13, Wiglaf displayed a systematic pattern of labial substitution for coronal fricatives, affricates and laterals at the left edge of words. For sake of simplicity, we hereafter make reference to this substitution as the “labial-left” pattern and loosely refer to the consonants it affects as coronal continuants, given the element of continuancy common to the fricative, affricate and lateral manners of articulation. After we describe these data with the necessary level of detail and rule out alternative analyses for the emergence of the labial-left pattern, we take the child’s lexicon as the primary source of explanation for the emergence of this pattern, building on earlier work by Fikkert and Levelt (2008).

### Labial-Left Pattern in Wiglaf’s Productions of Coronal Continuants

As we can see in the examples in (1a), Wiglaf was perfectly able to produce labial stops and continuants, both before and throughout the labial-left period (1;08.02 to 1;10.13). Similarly, in (1b), Wiglaf was able to produce coronal stops at the left edge of words, also from the beginning of the observation period.

(1) Wiglaf’s word-initial labial stops and continuants and coronal stops.

**Table:** 


a. Labial stops and continuants.				
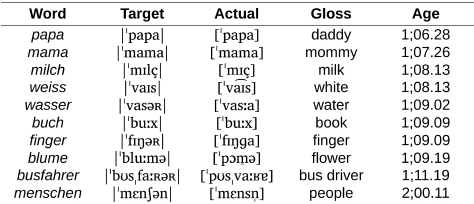				

**Table:** 


b. Coronal stops.				
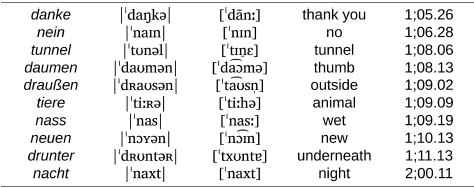				

**FIGURE 4:**
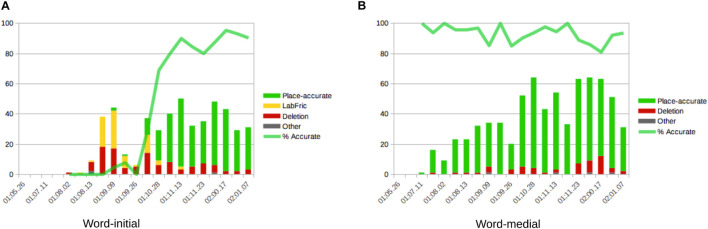
Wiglaf’s development of coronal continuants in syllable onsets. **(A)** Word-initial. **(B)** Word-medial.

However, as we can see in Figure 4A, the corpus records the first attempt at words with coronal continuants only at 1;07.26, approximately 4 months after the beginning of the documentation period for this child. From there, between 1;08.02 and 1;10.13, Wiglaf produced coronal continuants accurately in only 14 out of 149 attempts (9.4%), with 11 of these accurate productions recorded within the very last transcript documenting this period. In comparison, we can see in Figure 4B that the child’s productions of coronal continuants in word-medial onsets were highly accurate throughout the observation period, with performance at a virtual ceiling from the first productions recorded in the corpus (211 out of 227 attempts, for a 93% place-accuracy rate), also without a single case of labial substitution attested in this position.

We exemplify the pattern of labial substitution affecting coronal continuants in initial syllables in the examples in (2a). Among other details, we can see through examples such as *lecker*, *lenken*, *zettel*, and *zehn* that labial substitution cannot be attributed to individual word shapes (it applies to both monosyllables and disyllables involving different consonants and clusters), nor to the presence of round vowels or other labial consonants within the word. As already noted, labial substitution also applied to affricates (e.g., *zahlen*, *zettel*), which the child optionally produced as fricatives throughout the observation period (e.g., *zimmer* |ˈʦɪməʀ| “room” produced as [ˈsɪm̠a] at 01.11.13; see Watts, 2018, pp. 126–127, for more detail about Wiglaf’s development of affricates). Together, these observations rule out analyses involving consonant harmony (Smith, 1973; Goad, 1996; Pater, 1997; Rose, 2000), consonant-vowel interactions (Levelt, 1994; Fikkert and Levelt, 2008), or potential effects related to syllable truncation. In contrast to this, Wiglaf’s ability to produce coronal continuants in word-medial onsets is exemplified in (2b). His labial substitution pattern was thus truly conditioned by an interaction between specific phonological categories in a specific position within the word.

(2) Wiglaf’s labial-left pattern affecting coronal continuants.

**Table:** 


a. Coronal continuants at the left edge of words.				
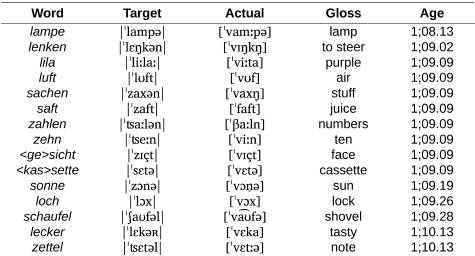				

**Table:** 


b. Coronal continuants in word-medial onsets: Accurate production.				
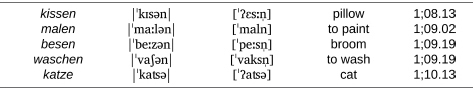				

**FIGURE 5:**
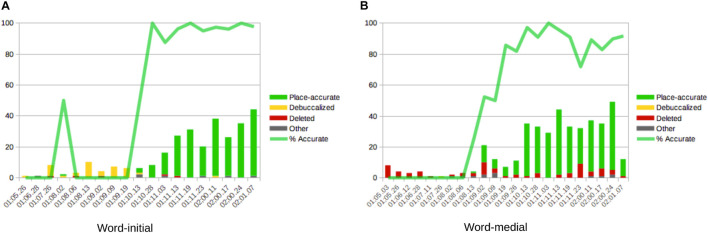
Wiglaf’s development of velars in syllable onsets. **(A)** Word-initial. **(B)** Word-medial.

For sake of exhaustiveness, in addition to the coronal and labial data described already, we observe Wiglaf’s early reluctance to attempt words which begin with velars consonants and his early inability to reproduce these consonants in his productions, across all positions within the word, as illustrated in Figure 5. During the stage of velar emergence, the leading pattern in word-initial position was that of debuccalization (to laryngeals [ʔ, h]), without any noticeable pattern of substitution to labials. In word-medial positions, the few target velars attempted by Wiglaf primarily underwent deletion.

Velars began to emerge in Wiglaf’s productions during the latter part of the labial-left stage described above, first in medial positions, at 1;09.02, and then in initial positions, at 1;10.13. In spite of the overlap between the emergence of velars and that of coronal continuants, we have no reason to think that these two developments are empirically or formally related. First, the patterns observed operate on different classes of sounds (coronal continuants vs. velar stops), and yield different outcomes (labial substitution vs. debuccalization to laryngeals). Second, Wiglaf’s development of velars does not display asymmetries between initial and medial positions. Finally, Wiglaf acquired velars at a slightly later stage than he acquired his coronal continuants in initial position. Overall, Wiglaf’s development of velars was in fact much more similar to that of his uvular rhotics, illustrated in Figure 3B, which he also mastered at 1;10.13, also after an initial stage marked by debuccalization. From a phonological perspective, this is consistent with the view that both velars and uvulars can be grouped under a single (dorsal) articulator. Wiglaf thus showed distinct patterns of phonological development across the three supralaryngeal places of articulation, with labial consonants and coronal stops acquired early and without noticeable difficulties, coronal continuants undergoing labial substitution at the left edge of words, and dorsal (velar and uvular) consonants undergoing debuccalization to laryngeals during their initial stages of emergence.

Any analysis of Wiglaf’s development of labials, coronals (stops and continuants) and velars should thus involve categories representing specific places and manners of articulation, also in reference to different prosodic positions. Each of these units and positions has its place in the general model of Figure 1. Whether the subsegmental levels are encoded in terms of articulatory gestures (e.g., Browman and Goldstein, 1989; Goldstein et al., 2007) or phonological features (Jakobson, 1941; Trubetzkoy, 1969; Smith, 1973; see Levelt, 1994; Bernhardt and Stemberger, 1998 for different feature-based analyses) is a debate which transcends the scope of this paper. A more immediate concern is the question as to why labials emerged as substitutes for the continuant class of coronals in Wiglaf’s productions. This substitution, which cannot be predicted on phonological or phonetic grounds alone, falls within the group of formally unexpected patterns that pervade the literature on child phonology (Priestly, 1977; Rose and Inkelas, 2011). However, this pattern is not exceptional in that it has been observed previously, in data on the acquisition of Dutch, a language which shares several lexical and phonological similarities with German. In the next subsections, we build on the original proposal by Fikkert and Levelt (2008), who first reported the occurrence of this pattern, and show how it can be explained through an emergentist approach which takes the full system as represented in Figure 1 into consideration. In particular, we focus on phonological pressures that can emerge from the content of the child’s own lexicon.

### Labial-Left Effect in the Acquisition of Dutch Phonology

In their study of the development of place of articulation in Dutch, Fikkert and Levelt (2008) report on a broadly similar labial-left pattern. At the time when they were beginning to differentiate consonant places of articulation within word forms, some of the children documented in Fikkert & Levelt’s corpus displayed a bias toward the production of labial consonants at the left edge of words, even for words whose target forms do not begin with a labial, as in the examples in (3) from Dutch-learning children Eva and Robin.

(3) Labial-left pattern in Dutch (data from the Dutch-CLPF corpus on PhonBank).

**Table:** 


a. In conjunction with a round (labial) vowel.				
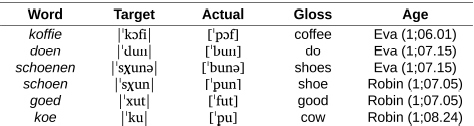				

**Table:** 


b. Independent of the presence of a round (labial) vowel.				
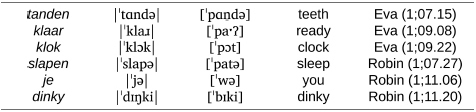				

Alongside these patterns, Fikkert and Levelt (2008) report on early speech productions patterns by children learning English, where the effects observed range from segmental substitution to metathesis (Ingram, 1974; Menn, 1983; Velleman, 1996), each of which reveal a bias toward labial-initial word forms. In the same vein, Garmann et al. (2019) report on a similar trend, based on a cross-linguistic comparison of Danish, English, Italian, Norwegian and Swedish acquisition data. Together, these observations suggest that labial-initial forms generally enjoy some privileged status, at least during the emergence of children’s earliest speech productive abilities^12^.

### Developmental Pressures From Speech Articulation

Building on Davis and MacNeilage (1995); Fikkert and Levelt (2008) suggest that physiological and motoric aspects of speech articulation make the production of labials inherently easier than that of other consonants in word-initial position (also, MacNeilage and Davis, 2000). In this view, labial articulations can be seen as a type of default speech articulation at the left edge of babbled forms, which has the potential to be phonologized as a preferred pattern by at least some children (Stoel-Gammon, 1989, 2011; McCune and Vihman, 2001). In turn, this preferred pattern can exert an influence on lexical development, yielding an early lexicon with a disproportionate number of labial-initial forms (see, also, Fikkert and Levelt (2008)). Vihman, 2014 argue that the labial-left bias they observe in their data can be traced directly to the early vocabularies of Dutch-learning children, as measured both through child-directed speech and the children’s own word selections, both of which involve a high prevalence of labial-initial words (see, also, van de Weijer, 1998; Dunphy, 2006). In sum, while articulatory biases are arguably universal, as they relate to basic mechanisms of speech production shared by all child speakers, these biases are more likely to be phonologized if they are reinforced by other components of the system, here the content of the child’s lexicon.

**FIGURE 6:**
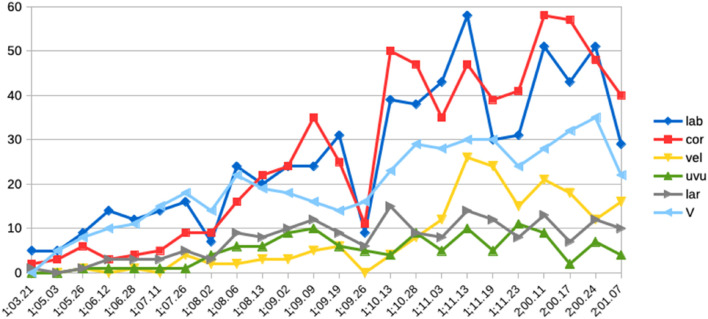
Wiglaf’s attempted word types, by initial sounds.

If we take the initial consonants of Wiglaf’s early attempted word forms as a proxy for the shape of his early lexicon, we obtain a very similar scenario. Figure 6 provides the number of individual words (word types) attempted by Wiglaf throughout the documentation period. As we can see, labial-initial words were clearly dominant in the child’s early lexical productions, alongside vowel-initial words, until 1;08.02. This age also corresponds to the child’s earliest attempts at words beginning with coronal fricatives and the emergence of the concomitant labial-left pattern, as we already saw in Figure 4.

**FIGURE 7:**
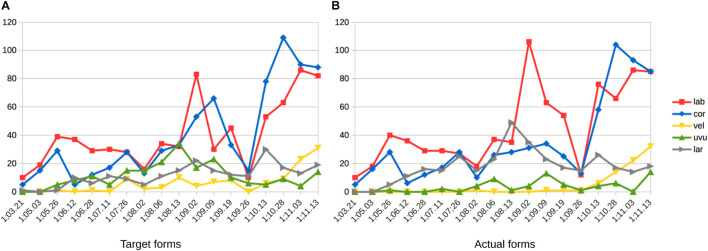
Wiglaf’s word-initial places of articulation (token) between 1;03.21 and 1;11.13. **(A)** Target forms. **(B)** Actual forms.

Turning now to the child’s actual word productions, Figure 7 displays the number of tokens for each word-initial consonant found in (a) target forms and (b) Wiglaf’s realizations of these forms. For clarity, the charts cover only the time period relevant to the present discussion, from 1;03.21 to shortly after the resolution of the labial-left pattern, at 1;10.13. Focusing first on the labial place of articulation, we can see in Figure 7A that words with initial labials were attempted the most often by the child.

This trend is matched in the actual data in Figure 7B, except for the disproportionate number of labial-initial forms in Wiglaf’s actual productions during the period marked by the labial-left pattern. Coronal-initial words then gradually took over, starting at 1;09.09, approximately 1 month before the resolution of the labial-left pattern at 1;10.13. These delayed effects between changes in the input to the child’s grammar and their manifestations through the child’s system, both during the period before the emergence of the labial-left pattern and during the period preceding its resolution, are also predicted by emergentism, given the time needed for the grammar to update itself based on changes in the input.

Finally, the remainder of the data in Figure 7 further substantiates the other developmental patterns noted above. This includes differences between the number of initial velars attempted by the child, in comparison to their rare occurrences in actual forms until 1;11.03. We also observe marked mismatches between the numbers of attempts and actual realizations of the uvular and laryngeal places of articulation. These mismatches come from Wiglaf’s early pattern of [h] substitution for |ʀ| already discussed in see section “Cross-Linguistic Survey on the Development of Rhotic Consonants” (see, also, Watts, 2018, pp. 129–130).

Recall, as we saw in Figure 4 and in the examples in (2), that Wiglaf’s labial substitutions at the left edge of words affected initial continuant coronals only. We attribute this to the fact that the child had more difficulties articulating this newly introduced class of sounds in word-initial position than, for example, labial or coronal stops. We take Wiglaf’s early difficulties with the production of coronal continuants in word-initial position, together with the prominence of the labial place of articulation in this position within his lexicon, as the primary sources of the pattern observed. As Wiglaf came to resolve production issues with coronal continuants in word-initial position, he then rapidly transitioned out of the substitution stage. However, while coronal stops would have seemed, from a phonetic standpoint, the most obvious substitutes for the coronal fricatives, the pattern of substitution to labials supports Fikkert and Levelt’s (2008) original proposal that the phonological properties of the child’s lexicon may condition patterns of development. Again here, neither a purely phonological nor a phonetically based analysis can capture the full set of observations; only a view of phonological emergence where every component of the system such as those represented in Figure 1 may potentially affect developmental patterns captures all the facts reported above.

## Discussion

The emergence of phonological productive abilities involves processing at various levels of lexical and phonological representation, with each of these levels highlighting the presence of different segmental categories and prosodic domains. In the context of our cross-linguistic survey of rhotic development, we emphasized that developmental differences observed between languages can be traced to both language-specific systems of contrasts and the phonetic expression of these contrasts in speech. Similarly, the labial substitution pattern affecting coronal continuants at the left edge of words in Wiglaf’s early productions can be related to general phonetic pressures, whose expression (through segmental substitution) can be traced directly to phonological properties of the learner’s own developing lexicon. While it is methodologically difficult to validate causal links between phonological patterning and properties of the child’s lexicon, the general proposal by Fikkert and Levelt (2008) which we embraced above offers compelling working hypotheses toward further research on the topic. Note in this regard that despite the commonalities between the labial-left patterns observed in both the German and the Dutch data, two very closely related languages, the current proposal does not predict that all learners of these (or other, similar) languages should necessarily display such intricate patterns of substitution. Yet, because these patterns are clearly attested in the data of at least some learners, we must maintain models of phonology and acquisition that allow us to capture them in meaningful ways, here in connection to the children’s developing lexicons. More generally, without a consideration of both small and larger units (here, phonological features and properties of word forms present in Wiglaf’s lexicon), alternative analyses of these data would likely be left without a clear hypothesis as to why the labial-left pattern emerged in the first place.

As an reviewer suggested to us, many different accounts of Wiglaf’s labial-left pattern could be formulated in constraint-based frameworks such as Optimality Theory (OT; also Bernhardt and Stemberger, 1998; Prince and Smolensky, 2004, for accounts of unusual patterns of phonological development within OT). These accounts, the exact formulation of which transcends the scope of this paper, provide useful insight into the functioning of phonological grammars, for example concerning tensions between phonological complexity and articulatory simplicity. However, these accounts are typically based on pre-existing phonological categories and constraints, whose origins are often not discussed within the literature, either on grounds that this topic is tangential to the issues at stake within individual papers or given commonly held assumptions about innateness. Consequently, these accounts provide rather limited grounds to investigate alternative views about the origins of phonological primitives^13^. In contrast to this, views of emergentism which impose no arbitrary limits on categorization have the potential to help demystifying the origins of linguistic categories central to representational approaches to phonology, also in ways which can remain fully compatible with current theories of phonology in most respects, of course besides nativist assumptions (Rose, 2014).

Wiglaf’s labial-left pattern must also be placed within the larger literature on relations between phonological development and that of the lexicon. Recall that the first word forms produced by individual children tend to emerge in accordance with the most prominent (or preferred) productive abilities expressed through their late babbles (Stoel-Gammon, 2011; Vihman, 2014). Recent research in this area also adds interesting subtlety to this observation, pointing at asymmetries between different places and manners of articulation across different prosodic positions (Davis et al., 2018). These asymmetries further corroborate the observations discussed above, whereby constraints on place of articulation appear to exert prominent influences on the word-initial consonants of children’s early word productions, while other positions (e.g., medial, final) do not seem to be constrained nearly to the same extent. However, studies of lexical development in older children point to other factors, including word meanings (Takac et al., 2017), especially at later stages when the child’s vocabulary development is no longer constrained by their own phonological productive abilities.

Observations such as these suggest different stages of emergence, during which the various components of the child’s system exert different levels of influence on developmental outcomes. Coming full circle with our introductory discussion, the nature of the acquisition data we considered in this paper, which focuses on the earliest stages of phonological development in production, currently prevents us from directly addressing the acquisition of phonological abstraction based on morpho-phonological alternations. Recall the cross-linguistic attestation of adult-language morpho-phonological patterns which transcend natural classes of sounds defined on phonetic grounds. The facts and analyses discussed above offer us a logical starting point, that children initially master phonological representations which are intimately connected to phonetic and phonological properties of speech. At later stages, as children begin to break into the system of morpho-phonological alternations of their language, they are then in a position to draw more abstract generalizations and adjust their phonological representations accordingly. In contexts where morpho-phonological alternations contradict expectations based on speech phonetics, the current view also predicts the potential emergence of error patterns reflecting these expectations. We leave the empirical exploration of this hypothesis for further research.

Finally, while emergentism offers many testable hypotheses about phonological development, the same cannot be said of approaches to phonology which assume an innate (and, thus, universal) set of representational primitives, given that these approaches can readily capture neither patterns of child phonology (Hale and Reiss, 2003, 2008) nor cross-linguistic variation in the phonological patterning in adult languages (Mielke, 2005b, 2013; Cowper and Currie Hall, 2014; Dresher, 2014, 2018). Similar issues, but for very different reasons, also undermine maximally concrete, word-based models of linguistic representation and processing. Given that these models either impose arbitrary limits on abstraction (e.g., Vihman and Croft, 2007), or reject the notion of categorical abstraction altogether (e.g., Ambridge, 2020), they are not equipped to capture, let alone explain, the types of segmental and/or positional observations highlighted throughout this article (Rose, 2020). More generally, by their very definition, these models would also fail to capture alternations relevant to adult phonological systems, let alone any segmental or sub-segmental effects these systems may have on acquisition. Until the debate has settled as to how much abstraction is ultimately needed to account for both the functioning of adult phonological systems and their acquisition, we contend that a consideration of all of the factors which may potentially emerge from different aspects of the learner’s (or speaker’s) system offers the most promising approach to further our understanding of all the relevant facts.

In sum, emergentist models which embrace multiple levels of phonological representation are best equipped to capture patterns of language development in relation to the properties of adult phonological systems. Within these models, each level of representation relevant to the functioning of the adult system emerges based on the evidence available to the learner at different points throughout the development process. These models thus offer compelling insights toward our understanding of both the nature and the origin of phonological knowledge. They also offer principled grounds to foster our understanding of how different components of the child’s developing system interact throughout the development process.

## Data Availability Statement

Publicly available datasets were analyzed in this study. This data can be found here: https://phonbank.talkbank.org.

## Author Contributions

YR was principally responsible for the text as well as the research on rhotic development. NP contributed the original observations about the labial-left pattern and all related data descriptions. Both authors provided their input to the methodological and theoretical discussions as well as to the final form of the document.

## Conflict of Interest

The authors declare that the research was conducted in the absence of any commercial or financial relationships that could be construed as a potential conflict of interest.

## Publisher’s Note

All claims expressed in this article are solely those of the authors and do not necessarily represent those of their affiliated organizations, or those of the publisher, the editors and the reviewers. Any product that may be evaluated in this article, or claim that may be made by its manufacturer, is not guaranteed or endorsed by the publisher.

## Footnotes

1 Throughout this article, we use “pipes” (|word|) to denote target, or model, forms for the child to acquire, which is formally different from phonological forms stored within the lexicon, conventionally represented between forward slashes (/word/), or the child’s actual renditions of these forms, enclosed between brackets ([word]).

2
https://phonbank.talkbank.org

3 The original Freitas corpus has since been reformatted into the Pereira-Freitas corpus. The results we present below are based on the original dataset.

4
https://www.phon.ca

5 This description excludes sC clusters, which show their own unique set of distributional properties (see Goad, 2016, for an extended discussion).

6 Portuguese displays a slightly more complex system, involving both a uvular and an apical rhotic, both of which are allowed in singleton onsets, with the added constraints that only the apical rhotic can appear in onset clusters and syllable codas, while only the uvular can appear in word-initial singleton onsets (Mateus and d’Andrade, 2000).

7 Rhotics in many dialects and idiolects of English are better described through tongue “bunching” (Scobbie et al., 2015).

8 Note as well that segmental deletion may also occur because of issues in prosodic structure development, similar to the word-initial syllable truncations discussed above. However, our primary focus on word-initial singleton onsets enables us to maximally avoid this additional confound.

9 Recall that this data compilation focuses on the word-initial context only. Additional variation is expected, both within and across languages, concerning non-initial positions (e.g., medial onsets, final codas). This issue, however, transcends the scope of the current discussion.

10 Note that [h] substitution for |ʀ| is fully independent from syllable truncation; in this respect, the laryngeal production in this and similar examples is by no means a reflex of the truncated syllable.

11 Patterns are more variable in post-vocalic position (at least in rhotic dialects of English), with [j] productions for |ɹ| more prominently found in this context (Smit, 1993). Again here, this variability in the child productions can be traced to auditory and related articulatory properties of |ɹ| (Ladefoged and Johnston, 2011, p. 94).

12 We might even speculate that the factors giving rise to the labial-left patterns are generally similar across all of these languages, a topic that would require additional explorations of early lexical development in each, also in comparison to that of other, phonotactically less similar languages.

13 See, however, Hayes (1999) on the origins of phonological constraints, which can also be related to phonetic and distributional properties of the ambient language, especially as represented within the learner’s own lexicon, a view largely compatible with our claims about phonological representations.

## References

[B1] Ambridge B. (2020). Against stored abstractions: a radical exemplar model of language acquisition. *First Lang.* 40 509–559. 10.1177/0142723719869731

[B2] Barlow J. A. (1997). *A Constraint-Based Account of Syllable Onsets: Evidence from Developing Systems* (Ph.D. Dissertation). Bloomington, IN: Indiana University.

[B3] Bernhardt B. H. Stemberger J. P. (1998). *Handbook of Phonological Development from the Perspective of Constraint-Based Nonlinear Phonology.* San Diego, CA: Academic Press.

[B4] Bernhardt B. M. Stemberger J. P. (2018). Tap and trill clusters in typical and protracted phonological development: conclusion. *Clin. Linguist. Phonet.* 32 563–575. 10.1080/02699206.2017.1370496 28956654

[B5] Bernhardt B. M. Másdóttir T. Stemberger J. P. Leonhardt L. Hansson G. Ó. (2015). Fricative acquisition in English- and Icelandic-speaking preschoolers with protracted phonological development. *Clin. Linguist. Phonet.* 29 642–665. 10.3109/02699206.2015.1036463 25985229

[B6] Browman C. P. Goldstein L. (1989). Articulatory gestures as phonological units. *Phonology* 6 201–251. 10.1017/S0952675700001019

[B7] Bybee J. L. (2001). *Phonology and Language Use.* Cambridge: Cambridge University Press.

[B8] Chomsky N. (1957). *Syntactic Structures.* The Hague: Mouton.

[B9] Chomsky N. Halle M. (1968). *The Sound Pattern of English.* New York, NY: Harper & Row.

[B10] Correia S. (2009). *The Acquisition of Primary Word Stress in European Portuguese* (Ph.D. Dissertation). Lisbon: University of Lisbon.

[B11] Correia S. da Costa T. Freitas M. J. (2010). *Corpus of European Portuguese Phonological Development.* Lisbon: Lisbon University/CLUL.

[B12] Cowper E. Currie Hall D. (2014). Reductio Ad Discrimen: where features come from. *Nordlyd* 41 145–164. 10.7557/12.3411

[B13] da Costa T. (2010). *The Acquisition of the Consonantal System in European Portuguese: Focus on Place and Manner Features* (Ph.D. Dissertation). Lisbon: University of Lisbon.

[B14] Davis B. L. MacNeilage P. F. (1995). The articulatory basis of babbling. *J. Speech Hear. Res.* 38 1199–1211. 10.1044/jshr.3806.1199 8747814

[B15] Davis B. Van Der Feest S. Yi H. (2018). Speech sound characteristics of early words: influence of phonological factors across vocabulary development. *J. Child Lang.* 45 673–702. 10.1017/S0305000917000484 29198209

[B16] Demuth K. (1995). “Markedness and the development of prosodic structure,” in *Proceedings of the North East Linguistic Society (NELS)*, Vol. 25 ed. Beckman J. N. (Amherst: Graduate Linguistic Student Association), 13–25. 10.1007/978-1-4757-5718-7_2

[B17] Demuth K. Tremblay A. (2008). Prosodically-conditioned variability in children’s production of French determiners. *J. Child Lang.* 35 99–127. 10.1017/S0305000907008276 18300431

[B18] dos Santos C. (2007). *Développement Phonologique en Français Langue Maternelle: Une Étude de Cas* (Ph.D. Dissertation). Lyon: Université Lumière Lyon 2.

[B19] Dresher B. E. (2014). The arch not the stones: universal feature theory without universal features. *Nordlyd* 41 165–181. 10.7557/12.3412

[B20] Dresher B. E. (2018). “Contrastive hierarchy theory and the nature of features,” in *Proceedings of the 35th West Coast Conference on Formal Linguistics*, eds Bennett W. M. G. Hracs L. Storoshenko D. R. (Somerville, MA: Cascadilla Press), 18–29.

[B21] Dunphy C. (2006). *Another Perspective on Consonant Harmony in Dutch* (M.A. Thesis). St. John’s, NL: Memorial University of Newfoundland.

[B22] Fikkert P. (1994). *On the Acquisition of Prosodic Structure.* The Hague: Holland Academic Graphics.

[B23] Fikkert P. Levelt C. C. (2008). “How does place fall into place? The Lexicon and emergent constraints in children’s developing grammars,” in *Contrast in Phonology: Theory, Perception, Acquisition*, eds Avery P. Dresher B. E. Rice K. (Berlin: Mouton de Gruyter), 231–268.

[B24] Freitas M. J. (1997). *Aquisição da Estrutura Silábica do Português Europeu* (Ph.D. Dissertation). Lisbon: University of Lisbon.

[B25] Garmann N. G. Hansen P. Simonsen H. G. Kristoffersen K. E. (2019). The Phonology of children’s early words: trends, individual variation, and parents’ accommodation in child-directed speech. *Front. Commun.* 4:10. 10.3389/fcomm.2019.00010

[B26] Gerken L. (1996). Prosodic structure in young children’s language production. *Language* 72 683–712. 10.2307/416099

[B27] Gnanadesikan A. E. (2004). “Markedness and faithfulness constraints in child phonology,” in *Constraints in Phonological Acquisition*, eds Kager R. Pater J. Zonneveld W. (Cambridge: Cambridge University Press), 73–108. 10.1017/cbo9780511486418.004

[B28] Goad H. (1996). “Consonant harmony in child language: evidence against coronal underspecification,” in *Proceedings of the UBC International Conference on Phonological Acquisition*, eds Bernhardt B. H. Gilbert J. Ingram D. (Somerville, MA: Cascadilla Press), 187–200.

[B29] Goad H. (2006). Are children’s grammars rogue grammars? Glide substitution in branching onsets. *Rech. Linguist. Vincennes* 35 103–132. 10.4000/rlv.1454

[B30] Goad H. (2016). “Phonological processes in children’s productions: convergence with and divergence from adult grammars,” in *The Oxford Handbook of Developmental Linguistics*, eds Lidz J. Snyder W. Pater J. (Oxford: Oxford University Press), 43–67.

[B31] Goad H. Rose Y. (2004). “Input elaboration, head faithfulness and evidence for representation in the acquisition of left-edge clusters in west Germanic,” in *Fixing Priorities: Constraints in Phonological Acquisition*, eds Kager R. Pater J. Zonneveld W. (Cambridge: Cambridge University Press), 109–157. 10.1017/cbo9780511486418.005

[B32] Goldstein B. A. (2007). “Spanish speech acquisition,” in *The International Guide to Speech Acquisition*, ed. McLeod S. (Clifton Park, NY: Thomson Delmar Learning), 539–553.

[B33] Goldstein L. Pouplier M. Chen L. Saltzman E. Byrd D. (2007). Dynamic action units slip in speech production errors. *Cognition* 103 386–412. 10.1016/j.cognition.2006.05.010 16822494 PMC2394196

[B34] Grimm A. (2006). “Intonational patterns and word structure in early child German,” in *Proceedings of the 30th Annual Boston University Conference on Language Development*, eds Bamman D. Magnitskaia T. Zaller C. (Somerville, MA: Cascadilla Press), 237–248.

[B35] Grimm A. (2007). *The Development of Early Prosodic Word Structure in Child German: Simplex Words and Compounds* (Ph.D. Dissertation). Potsdam: Universität Potsdam.

[B36] Hale M. Reiss C. (2003). The subset principle in phonology: why the tabula can’t be rasa. *J. Linguist.* 39 219–244. 10.1017/s0022226703002019

[B37] Hale M. Reiss C. (2008). *The Phonological Enterprise.* Oxford: Oxford University Press.

[B38] Hayes B. (1999). “Phonetically driven phonology: the role of optimality theory and inductive grounding,” in *Functionalism and Formalism in Linguistics: General Papers*, Vol. 1 eds Darnell M. Moravcsik E. Newmeyer F. J. Noonan M. Wheatley K. (Amsterdam: Benjamins), 243–285. 10.1075/slcs.41.13hay

[B39] Ingram D. (1974). Fronting in child phonology. *J. Child Lang.* 1 233–241. 10.1017/s0305000900000672

[B40] Inkelas S. (2003). J’s rhymes: a longitudinal case study of language play. *J. Child Lang.* 30 557–581. 10.1017/S0305000903005646 14513468

[B41] Jakobson R. (1941). *Kindersprache, Aphasie, und Allgemeine Lautgesetze.* Uppsala: Almqvist & Wiksell.

[B42] Jusczyk P. W. Houston D. M. Newsome M. (1999). The beginnings of word segmentation in English-learning infants. *Cognit. Psychol.* 39 159–207. 10.1006/cogp.1999.0716 10631011

[B43] Kleber F. (2018). VOT or quantity: what matters more for the voicing contrast in german regional varieties? Results from apparent-time analyses. *J. Phonet.* 71 468–486. 10.1016/j.wocn.2018.10.004

[B44] Ladefoged P. Johnston K. (2011). *A Course in Phonetics*, 6th Edn. Boston, MA: Wadsworth, Cengage Learning.

[B45] Ladefoged P. Maddieson I. (1996). *The Sounds of the World’s Languages.* Cambridge, MA: Blackwell.

[B46] Leroy-Collombel M. Mathiot E. Morgenstern A. (2009). “Pointing gestures and demonstrative words: deixis between the ages of one and three,” in *Studies in Language and Cognition*, eds Zlatev J. Johansson Falck M. Lundmark C. Andrén M. (Cambridge: Cambridge Scholars Publishing), 386–404.

[B47] Levelt C. C. (1994). *On the Acquisition of Place.* The Hague: Holland Academic Graphics.

[B48] Lin Y. Mielke J. (2008). Discovering place and manner features: what can be learned from acoustic and articulatory data. *Univ. Pa. Work. Pap. Linguist.* 14 241–254.

[B49] Lisker L. Abramson A. S. (1964). A cross-language study of voicing in initial stops: acoustical measurements. *Word* 20 384–422. 10.1080/00437956.1964.11659830

[B50] Macken M. A. Barton D. (1980). The acquisition of the voicing contrast in english: a study of voice onset time in word-initial stop consonants. *J. Child Lang.* 7 41–74. 10.1017/S0305000900007029 7372738

[B51] MacNeilage P. F. Davis B. L. (2000). On the origin of internal structure of word forms. *Science* 288 527–531. 10.1126/science.288.5465.527 10775113

[B52] Maddieson I. (1984). *Patterns of Sounds.* Cambridge, MA: Cambridge University Press.

[B53] Mateus M. H. d’Andrade E. (2000). *The Phonology of Portuguese.* Oxford: Oxford University Press.

[B54] Mattys S. L. Jusczyk P. W. Luce P. A. Morgan J. L. (1999). Phonotactic and prosodic effects on word segmentation in infants. *Cognit. Psychol.* 38 465–494. 10.1006/cogp.1999.0721 10334878

[B55] McAllister Byun T. Inkelas S. Rose Y. (2016). The A-map model: articulatory reliability in child-specific phonology. *Language* 92 141–178. 10.1353/lan.2016.0000 34409987

[B56] McCarthy J. J. Prince A. S. (1986). “Prosodic Morphology,” in *The Handbook of Phonological Theory*, ed. Goldsmith J. A. (Oxford: Blackwell), 318–366.

[B57] McCune L. Vihman M. M. (2001). Early phonetic and lexical development: a productivity approach. *J. Speech Language Hear. Res.* 44 670–684. 10.1044/1092-4388(2001/054) 11407570

[B58] McLeod S. Crowe K. (2018). Children’s consonant acquisition in 27 languages: a cross-linguistic review. *Am. J. Speech Lang. Pathol.* 27 1546–1571. 10.1044/2018_AJSLP-17-0100 30177993

[B59] Menn L. (1983). “Development of articulatory, phonetic, and phonological capabilities,” in *Language Production*, Vol. 2 ed. Butterworth B. (London: Academic Press), 1–49.

[B60] Menn L. Peters A. M. Rose Y. (2021). The Menn phonetic mini-corpus: articulatory gestures as precursors to the emergence of segments. *Front. Psychol*. 12:646090. 10.3389/fpsyg.2021.646090 33995202 PMC8113676

[B61] Menn L. Schmidt E. Nicholas B. (2009). Conspiracy and sabotage in the acquisition of phonology: dense data undermine existing theories, provide scaffolding for a new one. *Lang. Sci.* 31 285–304. 10.1016/j.langsci.2008.12.019

[B62] Menn L. Schmidt E. Nicholas B. (2013). “Challenges to theories, charges to a model: the linked-attractor model of phonological development,” in *The Emergence of Phonology: Whole-word Approaches and Cross-linguistic Evidence*, eds Vihman M. M. Keren-Portnoy T. (Cambridge: Cambridge University Press), 460–502. 10.1017/cbo9780511980503.022

[B63] Mielke J. (2005a). Ambivalence and ambiguity in laterals and nasals. *Phonology* 22 169–203. 10.1017/s0952675705000539

[B64] Mielke J. (2005b). Modeling distinctive feature emergence. *Proc. West Coast Conf. Formal Linguist.* 24 281–289.

[B65] Mielke J. (2008). *The Emergence of Distinctive Features.* Oxford: Oxford University Press.

[B66] Mielke J. (2013). “Phonologization and the typology of feature behavior,” in *Origins of Sound Change: Approaches to Phonologization*, ed. Yu A. C. L. (Oxford: Oxford University Press), 165–180. 10.1093/acprof:oso/9780199573745.003.0008

[B67] Morgenstern A. Parisse C. (2007). Codage et interprétation du langage spontané d’enfants de 1 à 3 ans. *Corpus* 6 55–78. 10.4000/corpus.922

[B68] Munson B. Edwards J. Beckman M. E. (2011). “Phonological representations in language acquisition: climbing the ladder of abstraction,” in *The Oxford Handbook of Laboratory Phonology*, eds Cohn A. C. Fougeron C. Huffman M. K. (Oxford: Oxford University Press), 288–309.

[B69] Munson B. Edwards J. Schellinger S. Beckman M. E. Meyer M. K. (2010). Deconstructing phonetic transcription: language-specificity, covert contrast, perceptual bias, and an extraterrestrial view of Vox Humana. *Clin. Linguist. Phonet.* 24 245–260. 10.3109/02699200903532524 20345255 PMC2941432

[B70] Ohala J. J. (1983). “The origin of sound patterns in vocal tract constraints,” in *The Production of Speech*, ed. MacNeilage P. F. (New York: Springer-Verlag), 189–216. 10.1007/978-1-4613-8202-7_9

[B71] Ostiguy L. Tousignant C. (1993). *Le Francais Québécois: Normes et Usages.* Montréal, QC: Guérin universitaire.

[B72] Pater J. (1997). Minimal violation and phonological development. *Lang. Acquis.* 6 201–253. 10.1207/s15327817la0603_2

[B73] Pierrehumbert J. B. (2003). Phonetic diversity, statistical learning, and acquisition of phonology. *Lang. Speech* 46 115–154. 10.1177/00238309030460020501 14748442

[B74] Priestly T. M. S. (1977). One idiosyncratic strategy in the acquisition of phonology. *J. Child Lang.* 4 45–65. 10.1017/s0305000900000477

[B75] Prince A. S. Smolensky P. (2004). *Optimality Theory: Constraint Interaction in Generative Grammar.* Cambridge, MA: Blackwell Publishing Ltd.

[B76] Rice K. (1993). A reexamination of the feature [sonorant]: the status of ‘Sonorant Obstruents.’ *Language* 69 308–344. 10.2307/416536

[B77] Richtsmeier P. T. (2010). Child phoneme errors are not substitutions. *Toronto Work. Pap. Linguist.* 33. Available at: https://twpl.library.utoronto.ca/index.php/twpl/article/view/6889

[B78] Roberts J. (2019). *Covert Contrast in the Acquisition of English /ɹ/: A Case Study* (M.A. Thesis). St. John’s, NL: Memorial University of Newfoundland.

[B79] Rose Y. (2000). *Headedness and Prosodic Licensing in the L1 Acquisition of Phonology* (Ph.D. Dissertation). Montreal, QC: McGill University. 10.13140/2.1.1793.3608

[B80] Rose Y. (2003). Place specification and segmental distribution in the acquisition of word-final consonant syllabification. *Can. J. Linguist.* 48 409–435. 10.1017/s0008413100000724

[B81] Rose Y. (2014). “The emergence of first language phonology: perception, articulation and representation,” in *New Directions in the Acquisition of Romance Languages: Selected Proceedings of the Romance Turn V*, eds Costa J. Fiéis A. Freitas M. J. Lobo M. Santos A. L. (Newcastle upon Tyne: Cambridge Scholars Publishing), 35–61.

[B82] Rose Y. (2020). There is no phonology without abstract categories: a commentary on Ambridge (2020). *First Lang.* 40 626–630. 10.1177/0142723720905908 PMC8851372 35185224

[B83] Rose Y. dos Santos C. (2010). “Stress domain effects in French phonology and phonological development,” in *Interactions in Romance: Selected Papers from the 38th Linguistic Symposium on Romance Languages*, eds Arregi K. Fagyal Z. Montrul S. A. Tremblay A. (Amsterdam: John Benjamins), 89–104. 10.1075/cilt.313.10ros PMC4877038 27227170

[B84] Rose Y. Inkelas S. (2011). “The interpretation of phonological patterns in first language acquisition,” in *The Blackwell Companion to Phonology*, eds Ewen C. J. Hume E. van Oostendorp M. Rice K. (Malden, MA: Wiley-Blackwell), 2414–2438.

[B85] Rose Y. MacWhinney B. (2014). “The PhonBank project: data and software-assisted methods for the study of phonology and phonological development,” in *The Oxford Handbook of Corpus Phonology*, eds Durand J. Gut U. Kristoffersen G. (Oxford: Oxford University Press), 380–401.

[B86] Rose Y. MacWhinney B. Byrne R. Hedlund G. J. Maddocks K. O’Brien P. (2006). “Introducing Phon: a software solution for the study of phonological acquisition,” in *Proceedings of the 30th Annual Boston University Conference on Language Development*, eds Bamman D. Magnitskaia T. Zaller C. (Somerville, MA: Cascadilla Press), 489–500. PMC4769870 26933382

[B87] Rose Y. McAllister T. Inkelas S. (2021). “Developmental phonetics of speech production,” in *Cambridge Handbook of Phonetics*, eds Setter J. Knight R.-A. (Cambridge: Cambridge University Press).

[B88] Scobbie J. M. Sebregts K. (2011). “Acoustic, articulatory and phonological perspectives on Rhoticity and /r/ in Dutch,” in *Interfaces in Linguistics: New Research Perspectives*, eds Folli R. Ulbrich C. (Oxford: Oxford University Press), 257–277.

[B89] Scobbie J. M. Gibbon F. E. Hardcastle W. J. Fletcher P. (1996). “Covert contrast as a stage in the acquisition of phonetics and phonology,” in *Papers Presented at Laboratory Phonology V: Acquisition and the Lexicon*, eds Broe M. B. Pierrehumbert J. B. (Cambridge: Cambridge University Press), 43–62.

[B90] Scobbie J. M. Lawson E. Nakai S. Cleland J. Stuart-Smith J. (2015). “Onset Vs. coda asymmetry in the articulation of English /r/,” in *Proceedings of the XVIIIth International Congress of Phonetic Sciences*, (Glasgow), 0704.

[B91] Selkirk E. O. (1980a). “Prosodic domains in phonology: Sanskrit revisited,” in *Juncture: A Collection of Original Papers*, eds Aronoff M. Kean M.-L. (Saratoga, CA: Anma Libri), 107–129.

[B92] Selkirk E. O. (1980b). The role of prosodic categories in English word stress. *Linguist. Inq.* 11 563–605.

[B93] Smit A. B. (1993). Phonologic error distribution in the Iowa-Nebraska articulation norms project: consonant singletons. *J. Speech Hear. Res.* 36 533–547. 10.1044/jshr.3603.533 8331911

[B94] Smith N. V. (1973). *The Acquisition of Phonology: A Case Study.* Cambridge: Cambridge University Press.

[B95] Stevens K. N. Keyser S. J. (2010). Quantal theory, enhancement and overlap. *J. Phonet.* 38 10–19. 10.1016/j.wocn.2008.10.004

[B96] Stoel-Gammon C. (1989). Prespeech and early speech development of two late talkers. *First Lang.* 9 207–223. 10.1177/014272378900900607

[B97] Stoel-Gammon C. (2011). Relationships between lexical and phonological development in young children. *J. Child Lang.* 38 1–34. 10.1017/S0305000910000425 20950495

[B98] Takac M. Knott A. Stokes S. (2017). What can neighbourhood density effects tell us about word learning? Insights from a connectionist model of vocabulary development. *J. Child Lang.* 44 346–379. 10.1017/S0305000916000052 26884360

[B99] Trubetzkoy N. (1969). *Principles of Phonology.* Berkeley, CA: University of California Press.

[B100] van Alphen P. M. Smits R. (2004). Acoustical and perceptual analysis of the voicing distinction in Dutch initial plosives: the role of prevoicing. *J. Phonet.* 32 455–491. 10.1016/j.wocn.2004.05.001

[B101] van de Velde H. van Hout R. (2001). *’r-atics: Sociolinguistic, Phonetic and Phonological Characteristics of /r/.* Bruxelles: Université Libre de Bruxelles.

[B102] van de Weijer J. (1998). *Language Input for Word Discovery.* Nijmegen: Radboud University Nijmegen.

[B103] Velleman S. L. (1996). “Metathesis highlights feature-by-position constraints,” in *Proceedings of the UBC International Conference on Phonological Acquisition*, eds Bernhardt B. H. Gilbert J. Ingram D. (Somerville, MA: Cascadilla Press), 173–186.

[B104] Vihman M. M. (2014). *Phonological Development: The First Two Years*, 2nd Edn. Hoboken: Wiley-Blackwell.

[B105] Vihman M. M. Croft W. (2007). Phonological development: toward a “radical” templatic phonology. *Linguistics* 45 683–725.

[B106] Waterson N. (1971). Child phonology: a prosodic review. *J. Linguist.* 7 179–211.

[B107] Watts E. (2018). *Markedness and Implicational Relationships in Phonological Development: A Longitudinal, Cross-linguistic Investigation* (Ph.D. Dissertation). St. John’s, NL: Memorial University of Newfoundland. 10.1080/17549507.2020.1842906 PMC7935768 33342295

[B108] Wiese R. (1996). *The Phonology of German.* Oxford: Clarendon Press.

[B109] Wiese R. (2003). The unity and variation of (German) /r/. *Z. Dialektol. Linguist.* 70 25–43.

[B110] Yamaguchi N. (2012). *Parcours D’acquisition des Sons du Langage Chez Deux Enfants Francophones* (Ph.D. Dissertation). Paris: Université Sorbonne Nouvelle Paris.

[B111] Yamaguchi N. (2015). L’acquisition phonologique, entre Jakobson et les modèles fréquentiels. *Langages* 198 31–49.

